# Effects of Poly(ADP-Ribose) Polymerase-1 Inhibition in a Neonatal Rodent Model of Hypoxic-Ischemic Injury

**DOI:** 10.1155/2017/2924848

**Published:** 2017-06-15

**Authors:** Melanie Klöfers, Jules Kohaut, Ivo Bendix, Josephine Herz, Vinzenz Boos, Ursula Felderhoff-Müser, Mark Dzietko

**Affiliations:** ^1^Department of Pediatrics I, Neonatology, University Hospital Essen, University Duisburg-Essen, Essen, Germany; ^2^Department of Neonatology, Charité-Universitätsmedizin Berlin, Berlin, Germany

## Abstract

**Background:**

Hypoxia ischemia (HI) to the developing brain occurs in 1–6 in 1000 live births. Large numbers of survivors have neurological long-term sequelae. However, mechanisms of recovery after HI are not understood and preventive measures or clinical treatments are not effective. Poly(ADP-ribose) polymerase-1 is overactivated in response to ischemia. In neonatal mice HI activates PARP-1 but its role in perinatal brain injury remains uncertain.

**Objective:**

Aim of this study was to explore the effect of TES448 (PARP-1-inhibitor) and hypothermia after an ischemic insult.

**Design and Methods:**

10-day-old Wistar rats underwent HI. TES448 was given 10 min, 3 hrs, and 6 hrs after hypoxia. Hypothermia was started 30 min after HI and brains were dissected at P12. Western blotting and histological staining were used to evaluate for degree of injury.

**Results:**

Protein expression of PARP-1 levels was diminished after TES448 treatment. Cresyl violet and TUNEL staining revealed decreased injury in male rat pups following TES448 and combined treatment. Female rats showed increased numbers of TUNEL-positive cells after combined therapy. TES448 inhibited microglia activation after hypoxic-ischemic injury. A cellular response including NeuN, Olig2, and MBP was not affected by PARP-1-inhibition.

**Conclusions:**

Inhibition of PARP-1 and hypothermia lead to an alteration of injury but this effect is sexually dimorphic.

## 1. Introduction

Despite significant progress in obstetrical and neonatal care, hypoxic-ischemic encephalopathy remains a leading cause of death and disability in children. In developed countries 1–6 per 1000 live-born children experience a hypoxic-ischemic insult to the brain during the neonatal period leading to significant life-long morbidity and mortality. Cerebral palsy, epilepsy, and visual impairment as well as cognitive and motor deficits are common neurological problems later in life [1, 2]. The pathogenesis of perinatal brain injury is complex, including gray and white matter structures to varying degrees, and depends on gestational age and developmental stage [3]. It has been shown in human imaging studies and animal models that damage to the brain does not only occur at the time of injury but continues to evolve over a period of days to weeks [3]. But the mechanisms behind this evolution of brain injury are not fully understood and the only recommended therapeutic intervention is hypothermia treatment [4, 5]. However, a substantial number of infants still suffer from neurological problems despite cooling therapy. Therefore, current research focuses on the development of neuroprotective strategies with potential add-on effects to hypothermia [6, 7]. Poly(ADP-ribose) polymerases are ubiquitously detectable in cerebral cell nuclei. They are activated in response to cellular stress and are involved in multiple nuclear mechanisms including DNA repair, regulation of transcription, cell division control, and cell death mechanisms [8–10]. PARP-1 is the most abundant isoform in the brain [11]. In pathological or stress conditions, PARP-1 is considerably increased and leads to different types of cell death including necrosis and caspase-independent mitochondrial membrane depolarization followed by the release of apoptosis-inducing factor (AIF) [12]. Furthermore, PARP-1 formation can influence transcription factors, notably nuclear factor kappa B, thereby promoting inflammation [13]. In the adult brain, PARP-1 contributes to neuronal injury and activation of microglia [14]. In a number of severe and acute diseases such as stroke, neurotrauma, circulatory shock, and acute myocardial infarction, activation of PARP-1 is detectable in human tissue samples supporting the clinical application of PARP-1 inhibition [14–16]. In the neonatal brain, it has been shown that hypoxia ischemia triggers PARP-1 activation [17, 18] and that disruption of the PARP-1 gene protects the developing brain predominantly in males [19]. Recently, the pharmacokinetics of a PARP-1 inhibitor (TES448, TES Pharma, Corciano, Italy) has been significantly improved and published data revealed a robust neuroprotective effect in an adult rat stroke model [20]. The aim of our study was to establish whether this PARP-1 inhibitor modulates hypoxic-ischemic brain injury in the* developing brain resembling the clinical situation of birth asphyxia in the term infant* [21, 22].

## 2. Materials and Methods

### 2.1. Ethics Statement

All animal procedures complied with the European Committee's Council Directive and were performed in accordance with the ethical guidelines of the University Duisburg-Essen and the German Animal Welfare Act. All procedures have been approved by the local animal welfare committees.

### 2.2. Experimental Protocol

Hypoxia ischemia (HI) was induced in 10-day-old Wistar rat pups through electrocoagulation (high temperature cautery, 1200°C, Bovie, USA) of the left common carotid artery under isoflurane anesthesia (2–5 Vol%). After surgery, animals were returned to their dams for a 60 min recovery period followed by 120 min of hypoxia (8% oxygen and balance nitrogen) conducted in an oxygen chamber (OxyCycler, Biospherix, USA). Body temperature during hypoxia was maintained through a warming mat (Harvard Apparatus, USA) set to 37°C. Sham controls underwent anesthesia and incision only. Weights were daily monitored for 48 hrs. Animals per litter and experiment were randomly attributed to treatment paradigms and experimenters were blinded of interventions and data analysis.* A total of 169 animals underwent the HI procedure including 82 males and 87 female rat pups. Animals were divided into following groups for histology: Sham + NaCl n* = 38*, HI + NaCl n* = 37*, HI + NaCl + HT n* = 21*, HI + TES448 n* = 30*, and HI + TES448 + HT n* = 16*. For immunoblotting animals were randomly selected as follows: Sham + NaCl n* = 8*, HI + NaCl n* = 8*, and HI + TES448 n* = 11.

### 2.3. Cooling Protocol

Hypothermia (HT) treatment was applied 30 min after end of hypoxia for the duration of 4 hrs. Therefore, sham-operated and ligated rats were placed on a custom-made hypothermia plate with temperature control by water circulation (28°C) with a goal of 32°C rectal temperature. Noncooled* rats* were kept with their dam. Body temperature was monitored in cooled and noncooled animals with a rectal probe for neonatal rodents (RET-4, Physitemp Instruments Inc., USA) connected to a digital thermometer (TH-5, Physitemp Instruments Inc., USA).* Measurements were performed in 6 to 8 sentinel animals per group right after end of hypoxia (0 min) and 60, 120, 180, and 240 min after end of hypoxia during cooling period. These measurements were done in all 4 treatment groups to ensure achievement of target rectal temperature or to monitor normal body temperature in the noncooled rats* (*Supplemental Figure *1 in Supplementary Material available online at https://doi.org/10.1155/2017/2924848).

### 2.4. Drug Administration Protocol

Animals were randomly assigned to treatment groups and received either normal saline or PARP-1 inhibitor (TES448). Before application, TES448 was dissolved in normal saline to obtain three different concentrations (0.3 mg/ml), stirred with vortex-mixing for 5 min at room temperature. The final solutions were filtered (syringe filter, 0.45 *µ*m* Supor*® membrane, PALL PharmAssure, Porsmouth, England). TES448 was given at the dose of 3, 10, and 20 mg/kg, intraperitoneally; 10 min, 3 hrs, and 6 hrs after the end of hypoxia. Normal saline served as the corresponding control and was injected at the same time points to sham or HI animals.

### 2.5. Histological Scoring

Animals were deeply anesthetized with chloral hydrate (200 mg/kg body weight) 48 hrs after end of HI and transcardially perfused with ice-cold phosphate buffered saline (PBS) followed by 4% paraformaldehyde (PFA, pH 7.4). After decapitation, brains were carefully removed, postfixed over night at 4°C, and then processed to be embedded in paraffin. Using a microtome (ThermoScientific, Walldorf, Germany), 10 *µ*m coronal sections were cut and mounted onto glass slides. To determine acute brain injury, adjacent paraffin sections were collected at the level of +0.2–+0.48 mm (striatum), −3.12–−3.36 mm (hippocampus), and −5.52–−5.76 mm from bregma (posterior cortex) and stained using cresyl violet. Brains were analyzed for degree of injury,* using 4 consecutive coronal sections at the striatal, hippocampal, and posterior cortical levels*, in a blinded fashion, using a scoring system, as previously described [23]. Briefly, 8 regions (anterior, middle, and posterior cortex, CA1, CA2, CA3, and dentate gyrus of the hippocampus, and striatum) were graded on a scale of 0 to 3 with 0 = no injury, 1 = few small areas of focal injury, 2 = multiples areas of focal injury, and 3 = widespread injury with loss of architecture. These 8 regional scores were summed to provide a scale of 0 to 24 where 0 denotes no injury and 24 denotes severe injury with cystic infarction.

### 2.6. Quantification of Cell Death

Degenerating cells were determined in 10 *µ*m thick paraffin sections collected at the hippocampal level (−3.12–−3.36 mm from bregma) using terminal deoxynucleotidyl transferase-mediated dUTP nick end labelling (TUNEL). Staining was performed using the In Situ Cell Death Detection Kit (Roche applied science, Penzberg, Germany) according to manufacturer's protocol. Sections were counterstained with 4,6-diamidino-2-phenylindole (DAPI, 1 *μ*g/mL, Invitrogen, Karlsruhe, Germany) and slides were mounted with Fluorescent Mounting Medium and kept in the dark at 4°C. Immunopositive cells were counted manually in three regions of interest in the parietal cortex and the entire hippocampus by an observer, unaware of the treatment protocol, with an Axioplan fluorescent microscope (Zeiss, Jena, Germany) at 20x (cortex) and 10x (hippocampus) magnification (Supplemental Figure 2).

### 2.7. Western Blotting

For protein analysis, animals were transcardially perfused with 0.1 M PBS 48 hrs after end of HI. After decapitation, brains were dissected, snap-frozen in liquid nitrogen, and stored at −80°C until analysis. Ipsi- and contralateral hemispheres were homogenized in ice-cold lysis buffer (RIPA, Sigma-Aldrich, Taufkirchen, Germany) containing protease, phosphatase inhibitors (cOmplete, Roche), and 100 mM PMSF (Sigma-Aldrich). Samples were centrifuged at 4°C for 10 min (3000 ×g) and the supernatant (cytoplasmic and mitochondrial fraction) was collected and recentrifuged at 17000 ×g for 20 minutes (4°C). The supernatant was collected (cytoplasmic fraction) followed by determination of the protein concentration using the Pierce BCA-protein assay kit (Thermo Scientific, USA). The pellet (nuclear fraction) was washed with cold PBS and again centrifuged at 3000 ×g for 10 minutes (4°C). The pellet (nuclear fraction) was rehomogenized with ice-cold lysis buffer (RIPA, Sigma-Aldrich) followed by disruption of the nucleus using an ultrasonicator (30 cycles each 30 sec with 30 sec pause). Then the fraction was recentrifuged at 17000 ×g for 20 min (4°C) and protein concentrations were determined in the supernatant. Forty micrograms of the protein lysates were heat denaturated in Laemmli sample loading buffer, separated by 8% sodium dodecyl sulfate polyacrylamide gel electrophoresis, and electrotransferred onto a nitrocellulose membrane (0.2 *µ*m, Amersham, USA) at 4°C overnight. Equal loading and transfer of proteins were confirmed by staining the membranes with Ponceau S solution (Fluka, Buchs, Switzerland). Nonspecific protein binding was blocked by incubation in 5% nonfat milk powder and 0.1% Tween in TBS (TBST), followed by incubation with the primary antibodies, anti-PARP (1 : 500; Santa Cruz Biotechnology, Heidelberg, Germany), anti-Olig2 (1 : 2500; Dako, Glostrup, Denmark), anti-NeuN (1 : 2000; Cell Signaling), anti-MBP (1 : 2000; Cell Signaling), anti-Lamin B2 (1 : 5000; Abcam plc, Cambridge, UK), and anti-GAPDH (1 : 1000, Santa Cruz Biotechnology). Membranes were then incubated with the appropriate peroxidase-conjugated secondary antibodies at room temperature for 1 hr (anti-rabbit 1 : 2000 or anti-mouse 1 : 5000, Dako, Denmark) in blocking solution followed by Chemiluminescent detection with the ECL prime Western blotting detection reagent (Amersham, GE Healthcare Life Science, USA). For visualization and densitometric analysis the ChemiDocXRS+ imaging system and ImageLab software (Bio-Rad, Germany) were used. All plots were normalized to GAPDH or Lamin B2 and animals were normalized to mean of the control group (Sham + NaCl).

### 2.8. Statistical Analysis

Analysis was performed using GraphPad Prism version 6.0 (GraphPad Software, La Jolla, CA, USA). All results were expressed as box plots including median values, the 25% and the 75% percentile. One-way ANOVA with Bonferroni's test post hoc analysis for multiple comparisons or unpaired Student's *t*-test as appropriate was performed for statistical analysis. Statistical significance was determined at *p* < 0.05.

## 3. Results

### 3.1. Weight Evolution after Unilateral HI and Dose Response of PARP-1 Inhibition

*TES448 at a dose of 3 mg/kg reduced injury scoring following HI. Higher doses (10 and 20 mg/kg) resulted in a lower degree or no protection after HI. All further experiments were conducted using 3 mg/kg (Supplemental Figure *3). At 48 hrs following HI, there was a significant difference of weight gain expressed by the ratio weight at P10/weight at P12 between HI and sham-operated animals. Those weight differences were not counteracted by PARP-1 inhibition or hypothermia treatment (Table 1). No abnormal behavior like convulsions and impaired or distinct activity was detected after TES448 administration.

### 3.2. PARP-1 Levels Are Affected by Inhibitor Therapy

**Figure 1:**
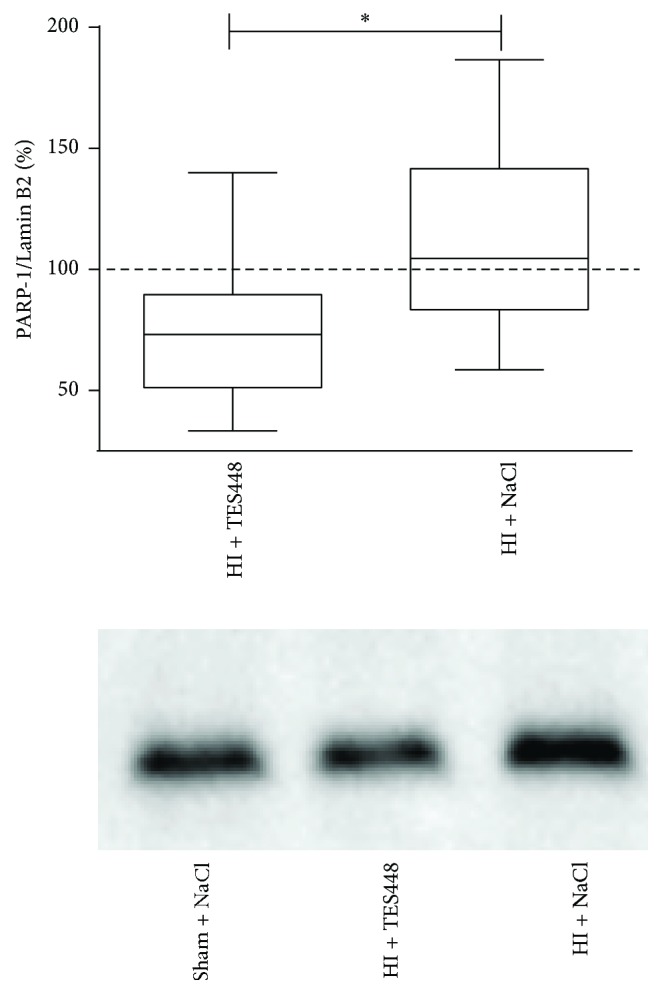
PARP-1 expression was analyzed in nuclear protein lysates using the entire hemisphere of animals exposed to HI followed by vehicle or TES448 treatment. A representative Western blot image shows the expression of PARP-1 at 48 hrs after HI. Data are normalized to sham animals (control = 100%, dashed line). Values are presented as box plots including median values, the 25% and the 75% percentile (^*∗*^*p* < 0.05, unpaired Student's *t*-test, *n* = 8–11 per group).

Cerebral PARP-1 levels were measured using Western blot to identify effects of HI and TES448 treatment on the developing brain. PARP-1 levels in the ipsilateral hemisphere were decreased in TES448 treated HI animals (74.3 ± 8.8) compared to saline treated HI animals (111 ± 14.4) 48 hrs after injury (Figure 1).

### 3.3. TES448 Treatment Modulates Histological Injury 48 hrs after Unilateral HI

**Figure 2:**
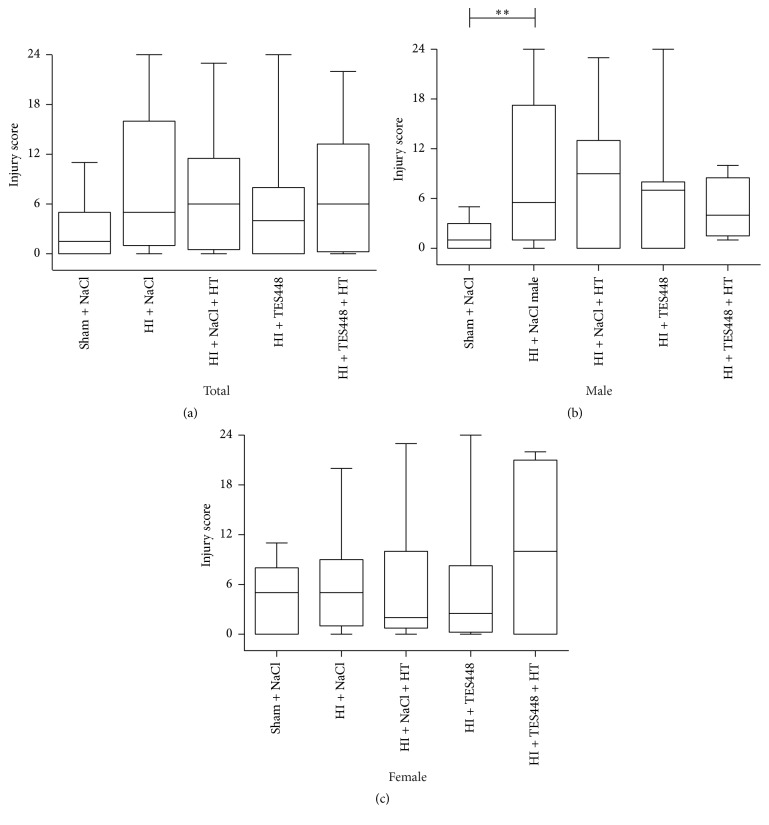
P10 rats subjected to HI, treated with saline or TES448 and either cooled or kept with their dam, were analyzed 2 days after HI to assess injury on cresyl violet stained sections. Values are presented as box plots including median values, the 25% and the 75% percentile, ^*∗∗*^*p* < 0.01, one-way ANOVA with Bonferroni's multiple-comparison test, total *n* = 16–38 per group, males *n* = 5–19 per group, and females *n* = 10–19 per group.

**Figure 3:**
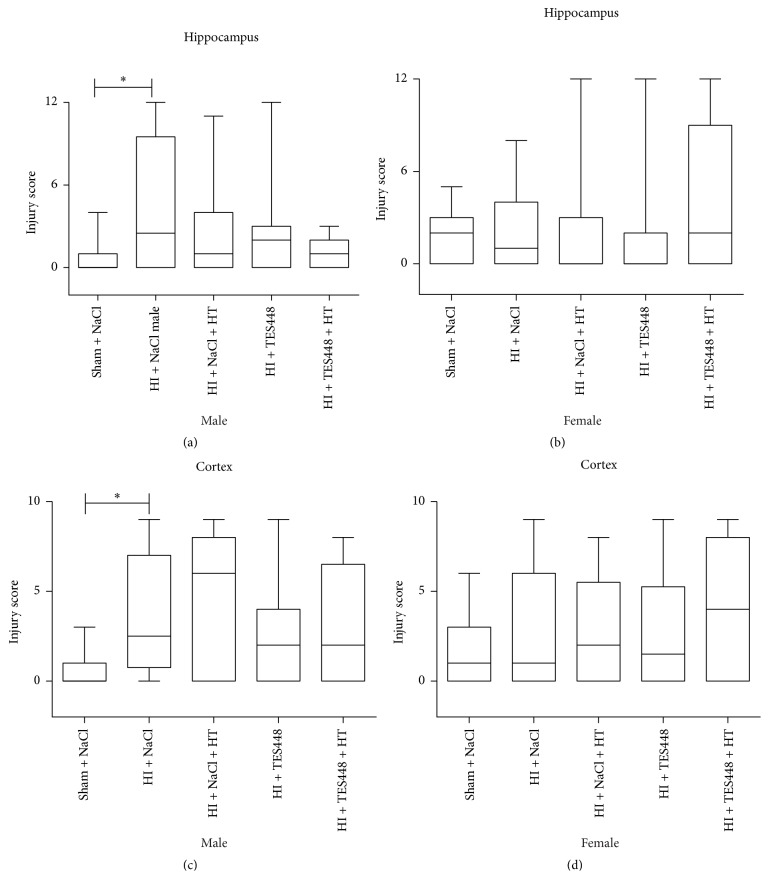
Histological analysis of brain regions (cortex and hippocampus) showed significant injury after HI. Hypothermia treatment (HT) or PARP inhibition (TES448) alone leads not to significant brain protection but a combined treatment (HT + TES448) reduced injury scoring in the hippocampus of male pups to the level of sham animals (a). In contrast, females were less severely injured after HI in the hippocampus but a combined treatment using hypothermia and TES448 augmented brain injury although no significant difference was detected (b). Values are presented as box plots including median values, the 25% and the 75% percentile, ^*∗*^*p* < 0.05, one-way ANOVA with Bonferroni's multiple-comparison test, males *n* = 5–19 per group, and females *n* = 10–19 per group.

Whether HT and PARP-1 inhibition provide early neuroprotection, brains were assessed by the use of an injury score. Forty-eight hrs after HI, histological changes were analyzed in the ipsilateral hemisphere using cresyl violet stained coronal sections (Figure 2). We detected increased brain injury predominantly in male animals exposed to HI and injected with saline (Figure 2(b)). PARP-1 inhibition, hypothermia treatment, or a combined therapy provided no significant protection of the developing brain (Figure 2).* In addition, female rat pups showed no significantly different injury scoring after a combined therapy of cooling and TES448 compared to sham controls suggesting a gender-specific sensitivity (Figure 2(c))*. A detailed analysis of brain regions (cortex and hippocampus) using histological scoring revealed significant injury in male pups to the cortex and hippocampus (Figures 3(a) and 3(c)). Hypothermia or PARP-1 inhibition alone preceded not to significant brain protection but a combined treatment reduced injury scoring in the hippocampus of male pups to the level of sham animals (Figure 3(a)).* Females showed no significant brain injury after HI in both regions of interest and a single or combined treatment using hypothermia and TES448 provided no significant histological recovery (Figures 3(b) and 3(d))*. Striatal tissue was not modulated in both genders by HI, HT, TES448, or a combined therapy (data not shown).

### 3.4. A Combined Therapy of PARP-1 Inhibition and Hypothermia Does Not Affect Apoptotic Cell Death in the Developing Rat Brain

**Figure 4:**
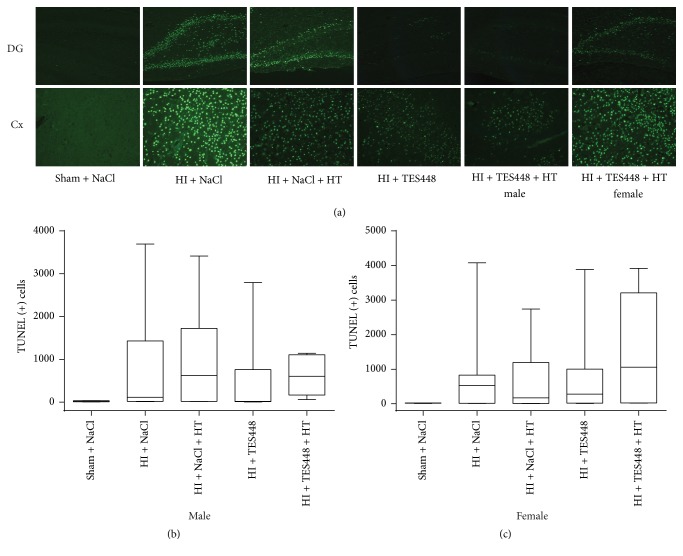
TUNEL staining revealed increased neurodegeneration after HI in the dentate gyrus (DG) and cortex (Cx) compared to sham controls receiving vehicle (a). Images of male animals receiving hypothermia (HT), TES448 only, or a combined therapy suggest a neuroprotective effect of PARP inhibition and cooling therapy after HI (a) although statistical significance could not be determined (b). Female rat pups demonstrated an increase of total TUNEL immunopositive cells after a combined treatment suggesting a gender-dependent effect of PARP inhibition and hypothermia ((a) and (c)). Values of total TUNEL-positive cells are presented as box plots including median values, the 25% and the 75% percentile, one-way ANOVA with Bonferroni's multiple-comparison test, males *n* = 5–11 per group, and females *n* = 7–11 per group.

In addition to cresyl violet staining we used TUNEL as a marker of DNA breakdown and apoptotic cell death to further evaluate degree of injury after TES448, cooling, or a combined therapy 48 hrs after end of hypoxia in P12 rat pups. Histological staining revealed increased neurodegeneration after HI in the cortex and hippocampus (Figure 4(a), HI + NaCl).* In age-matched sham littermates receiving vehicle, degenerating cells were sparse (Figure 4(a), Sham + NaCl). Imaging data suggest a reduction of TUNEL-positive cells following PARP-1 inhibition and a combined therapy after HI in the dentate gyrus (DG) in male animals (Figure 4(a), HI + TES448 and HI + TES448 + HT). Nevertheless, significance could not be determined following a combined quantification in cortex and the entire hippocampus (Figure 4(b)) in male rat pups. Furthermore, imaging data of TUNEL-positive cells in female rat pups demonstrated an increase of TUNEL-positive cells after a combined treatment (Figure 4(a), HI + TES448 + HT female) suggesting a gender-dependent effect of PARP-1 inhibition and hypothermia but a significant difference between males and females could not be determined in the HI + TES448 + HT group (TUNEL (+) cells total: male: 630, n* = 5* versus female: 1421, n* = 12*; p* = 0.29*, one-way ANOVA with Bonferroni's multiple-comparison test)*.

### 3.5. TES448 Induced PARP-1 Inhibition Modifies Cell Specific Changes after Unilateral HI

**Figure 5:**
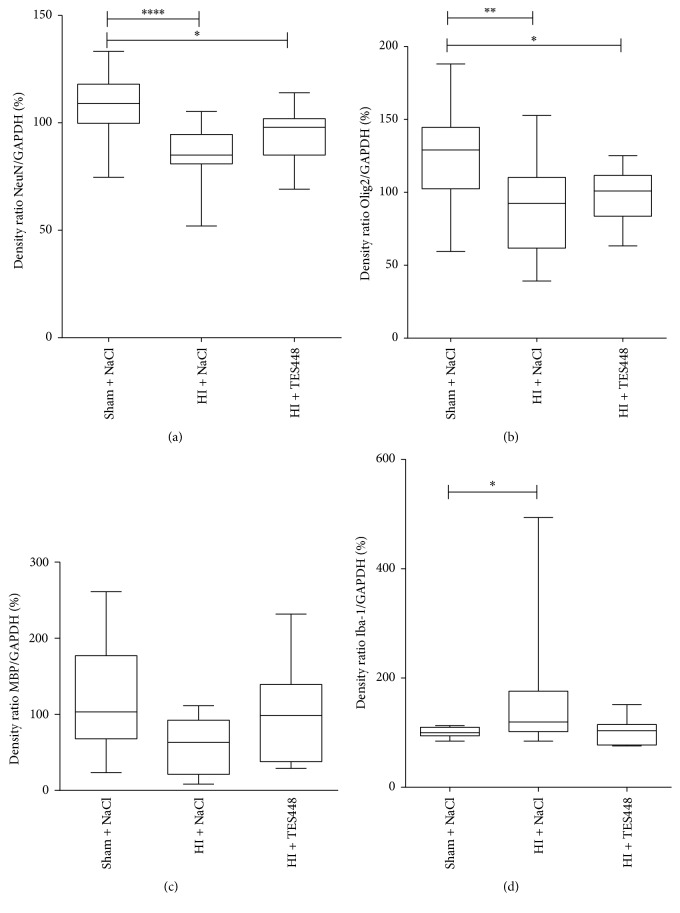
Protein expression of NeuN (a), Olig2 (b), MBP (c), and Iba-1 (d) was analyzed in protein lysates using the entire hemisphere of animals exposed to HI followed by TES448 treatment. Hypoxic-ischemic injury triggered a decrease of NeuN and Olig2 but additional TES448 therapy had no impact on protein expression. MBP expression was not affected by HI or PARP inhibition. Iba-1 expression was increased in hypoxic-ischemic animals injected with NaCl compared to sham control pups. Animals receiving TES448 treatment after HI reached the level of sham animals (d). Results are expressed as box plots including median values, the 25% and the 75% percentile, ^*∗*^*p* < 0.05, ^*∗∗*^*p* < 0.01, ^*∗∗∗∗*^*p* < 0.0001 one-way ANOVA with Bonferroni's multiple-comparison test, and *n* = 8–11 per group).

To more specifically differentiate cell types most affected by HI and PARP-1 inhibition, we performed Western blotting of NeuN, Olig2, MBP, and Iba-1 as markers for neurons, oligodendrocytes, and microglial activation. HI induced a detrimental reduction of neuronal and oligodendrocytic markers in the ipsilateral hemisphere 48 hrs after hypoxia compare to sham animals. TES448 was unable to counteract this effect efficiently (Figures 5(a) and 5(b)). MBP expression was not affected by HI or PARP-1 inhibition (Figure 5(c)). The expression of Iba-1 protein was used as a reflection of brain inflammatory response to unilateral HI. Forty-eight hrs after hypoxia, we observed an increased level of Iba-1 expression in the ipsilateral cerebral hemisphere of animals injected with NaCl compared to sham control pups. Animals receiving TES448 treatment after HI reached the level of sham animals suggesting a modulatory effect of PARP-1 inhibition on Iba-1 expression (Figure 5(d)).

## 4. Discussion

*In our model of severe brain damage, we confirm that PARP-1 inhibition influences hypoxic-ischemic injury in P10 male rats, modelling the clinical situation of birth asphyxia in the term infant [21, 22]*. This effect is sex-specific and preferentially protects male rat pups following HI. In addition, we describe that a* single* or combined therapy of hypothermia and TES448 is insufficient in female rats to enhance histological recovery.* Our data are in general agreement that PARP-1 levels are modified following ischemic injury in young and older animals [9, 11, 24]*. It has been reported that different types of PARP inhibitors or a disruption of the gene were able to promote significant recovery in ischemic brain injury supporting the hypothesis that brain injury in the immature brain depends on PARP-1 activation [17–20]. This effect seems to be sexually dimorphic with male animal's being protected and with female rodent's showing reduced protection after hypoxia ischemia [19, 20, 25, 26].* Similar to others hypothermia alone conducted for 4 hrs did not demonstrate neuroprotective effects in our model of severe injury [27–29]. This might be due to differences in mitochondrial physiology or different DNA repair polymerases in females compared to males. In addition, classical caspase-dependent apoptosis appears to be more prevalent in females, who therefore receive greater neuroprotective benefit from caspase inhibitors. The predilection for caspase-independent cell death in males, as in PARP-1 inhibition, may be due to decreased antioxidant defenses and increased susceptibility to ROS and peroxynitrite production, stimulating both mitophagy and parthanatos [30]*. Nevertheless, protection induced by hypothermia is a complicated, not completely understood process and consensus on the best protocol in rodents still needs to be determined. Experimental studies showed that hypothermia induced immediately after moderate HI generates protection [29, 31] but other groups provided evidence that hypothermia itself does not promote recovery following severe HI [32]. A previous study in P10 mice with HI showed that male animals are protected after hypothermia, while females had variable degrees of injury and protective effects could not be observed [27]. In addition, a combination of miscellaneous drugs with hypothermia revealed conflicting results regarding improved outcome after HI [33, 34], demonstrating that limiting multiple pathophysiological mechanisms may be needed to provide benefits. Furthermore, our results illustrate that a combined therapy provides no additional benefit and a simultaneous application may be used with caution if it is translated into clinical settings.* In our opinion, future research should focus on stratification of degree of injury, with treatment adjusted accordingly. With further investigation, it may turn out that animals with severe encephalopathy would derive greater benefits from drug treatments compared to hypothermia. One potential limitation of the present study is that due to technical limitations detrimental effects of hypoglycemia or a stress response to cooling could not be measured or prevented raising the issue that potential compensatory responses to cooling might interfere with the degree of injury*. Furthermore, short-term survival was analyzed only but in comparison to the clinical setting; long-term improvements in neurological outcome are the goal.* In the used HI model, certain short-term neuropathological markers do not always predict long-term injury [28] and behavioral improvement after neuroprotective treatment can be seen that are not reliably predicted by histological injury [35]*. As PARP-1 inhibition did not sufficiently reduce histological damage after HI, we analyzed specific brain regions known to respond differently [36] and to be selectively vulnerable to HI [37]. Degree of injury and protection by PARP-1 inhibition following HI was most pronounced in the hippocampus in male animals although the protective effect appears to be less robust than in adult HI models. In contrary, the hippocampus of females was not severely damaged following HI suggesting that mechanisms that have been proposed to explain neuroprotection obtained with PARP-1 inhibitors are not regional equally expressed in both genders. It has been shown that DNA lesions activate PARP-1 and cause excessive PAR accumulation, the product of PARP-1 activity, and parallel the time course of DNA damage following hypoxia ischemia in the brain [38]. Imaging data using TUNEL staining reflecting DNA breakdown depict a selective effect of PARP-1 inhibition in male rat pups emphasizing that male animals solely may benefit from treatment. Studies have provided evidence that molecular mechanisms in response to activation of PARP after experimental stroke are not identical in males and females probably explaining selectivity of PARP inhibitors [27]. It has been proposed that PARP-1-inhibitors are able to interact with all cell types of the neurovascular unit including the blood-brain barrier and to provide a better outcome compared to other neuroprotective strategies so far tested [9]. Therefore, we analyzed different cell types following HI and provide evidence that the cellular response after PARP-1 inhibition is diverse compared to the adult brain. We confirmed a previously describe attenuation of NeuN and MBP [31] after HI but TES448 was unable to counteract these effects suggesting a different sensitivity of the developing brain to PARP inhibition. The mechanism by which PARP-1-inhibitors suppress inflammatory responses after brain injury is not fully discovered but is likely to involve regulation of proinflammatory transcription factors [39–41] and microglial activation [12, 13]. Studies comparing juvenile and neonatal mice revealed a stronger inflammatory response in neonatal pups following HI [36]. However, recent experimental work using minocycline, a putative PARP inhibitor, inducing microglial suppression revealed an age-dependent response with neonatal animals showing improved histological and behavioral outcome compared to older animals [42]. Similarly, expression of Iba-1, a microglial marker, was prevented in our animals treated with TES448 suggesting a contribution of cerebral inflammation in the protective effects of PARP-1 inhibition. A recent review showed that immunomodulatory therapies attenuating neuroinflammation provide effectivity in experimental models supporting the use of immunomodulating therapies in neonatal HI to prevent neuronal injury [43]. Taken together, our results reveal that differences exist between male and female brains after exposure to ischemic injury and PARP-1 inhibition. These findings highlight the challenges testing combined therapies but also provide an opportunity for further investigation of therapy to prevent injury from HI.

## Supplementary Material

Figure 1: Measurement of rectal temperature following HI.
Figure 2: Regions of interest for counting of TUNEL positive cells.
Figure 3: Dose –response of PARP inhibition by TES448 in the neonatal rat brain.

## Figures and Tables

**Table 1:** Weight evolution after unilateral HI and TES448 treatment (HI versus sham controls, ^#^*p* < 0.0001, ^‡^*p* < 0.001, ±SEM, and one-way ANOVA with Bonferroni's multiple-comparison test).

	Sham + NaCl	Sham + TES448	HI + NaCl	HI + NaCl + HT	HI + TES448	HI + TES448 + HT
Weight at P10 (g)	20.42 ± 0.5	20.99 ± 0.5	21.08 ± 0.4	24.5 ± 1.0	21.32 ± 0.8	25.6 ± 1.0
Weight at P12 (g)	24.78 ± 0.5	25.42 ± 0.9	21.09 ± 0.6	25.5 ± 1.6	21.82 ± 0.6	26.1 ± 1.4
Gained weight	122% ± 1.0	122.3% ± 2.1	100%^#^ ± 2.0	104.1%^‡^ ± 4.1	103.4%^#^ ± 2.7	102.3%^#^ ± 4.5
