# Supplementary material for: Effects of Poly(ADP-Ribose) Polymerase-1 Inhibition in a Neonatal Rodent Model of Hypoxic-Ischemic Injury
Source: Biomed Res Int. 2017 Jun 15;2017:2924848. doi: 10.1155/2017/2924848 (PMC5494065; doi:10.1155/2017/2924848)
Supplement: Supplementary file 1 — Figure 1: Measurement of rectal temperature following HI. Figure 2: Regions of interest for counting of TUNEL positive cells. Figure 3: Dose –response of PARP inhibition by TES448 in the neonatal rat brain. [file 2924848.f1.pdf]

Supplement Figure S1

Effects of Poly-ADP-(Ribose-)-Polymerase-1 (PARP-1) inhibition in a neonatal rodent model of hypoxic-ischemic injury

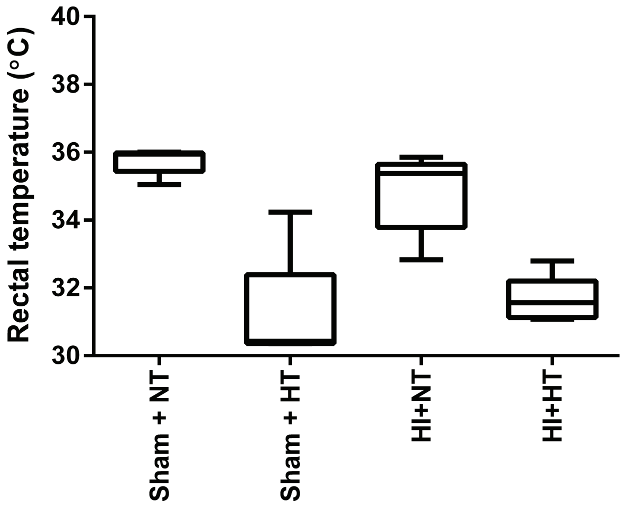

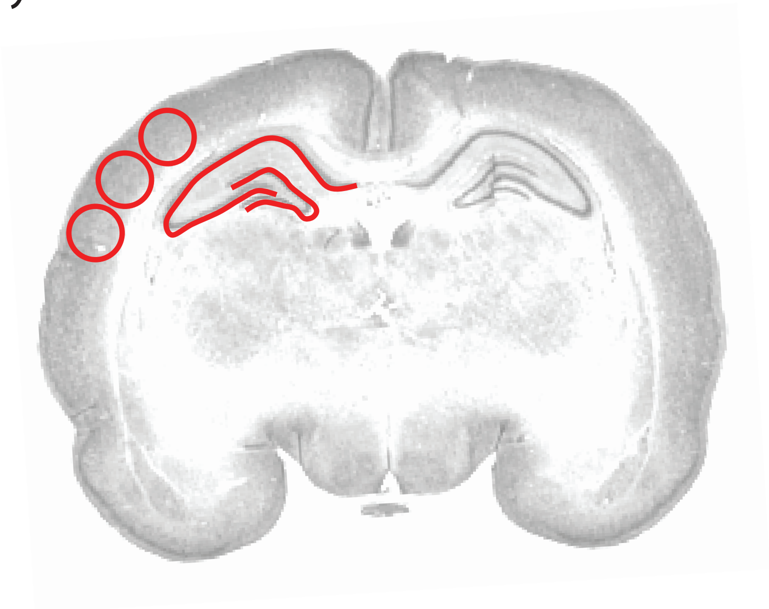

Figure 1: Measurement of rectal temperature at 120 min following HI revealed drop of body core temperature to 32 °C in hypothermia treated animals (n=6-8 sentinel animals per group).

Figure 2: TUNEL positive cells were counted manually in three regions of interest in the parietal cortex and in the entire hippocampus matching corresponding contralateral tissue serving as internal controls. Red circles represent regions in the core of the cortical injury.

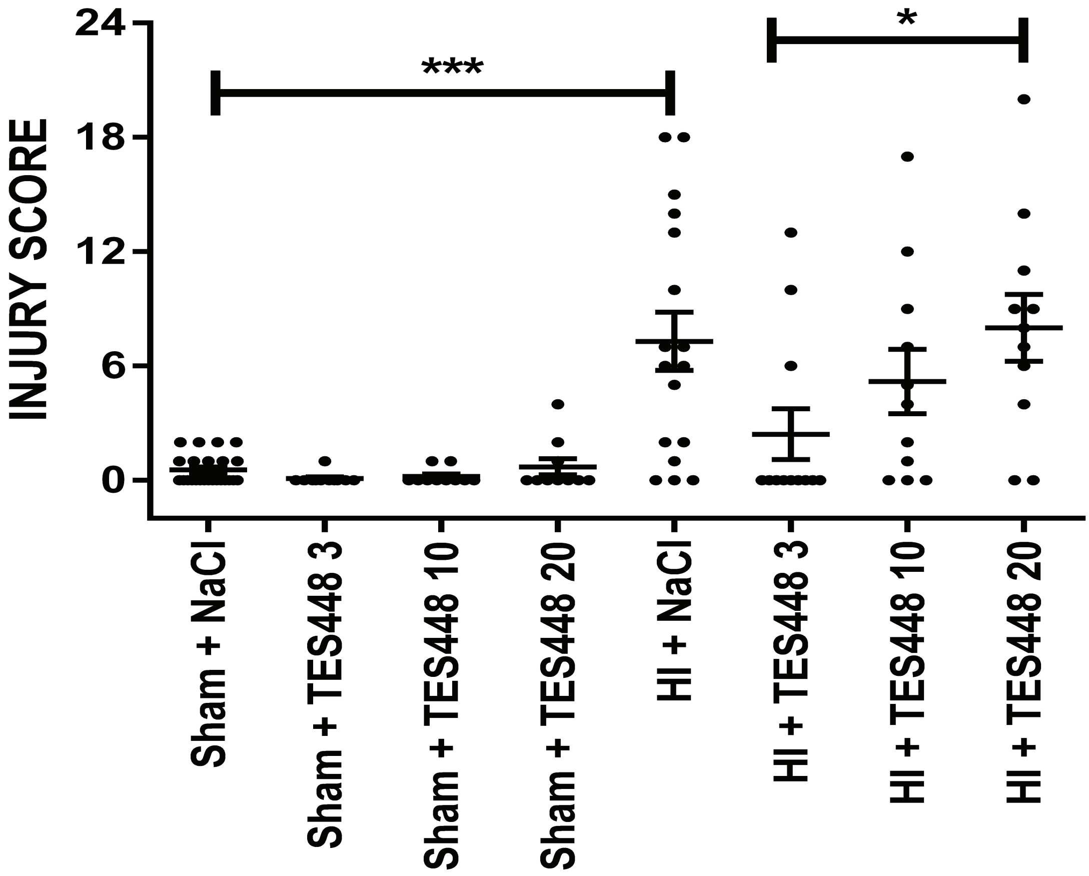

Figure 3: Dose –response effect of TES448 in the neonatal rat brain. P10 rats received three consecutive doses of TES448 (3, 10 or 20 mg/kg at 10 min, 3 and 6 hours) following HI. Histographic values represent cumulative injury scores in the forebrains of treated rats. Sham treated animals served as controls. (Values are presented as dot plots including mean values with SEM,*p<0.05, ***p<0.01, one-way ANOVA with Bonferroni's multiple-comparison test, n = 10-24 per group).
